# Fascia lata autograft for recurrent inguinal hernia repair in a geriatric pet rabbit (*Oryctolagus cuniculus*): a case report

**DOI:** 10.3389/fvets.2026.1913360

**Published:** 2026-09-22

**Authors:** Lucia Victoria Bel, Marcus Ofrim, Alexandra Creț, Iulia Melega, Ștefana Maria Cristina Mureșan, Romelia Pop, Robert Purdoiu, Teodora Patrichi, Ioana Madalina Moraru, Maria-Carmen Turcu

**Affiliations:** 1 New Companion Animals Clinic, Department of Surgery and ICU, Faculty of Veterinary Medicine, University of Agricultural Sciences and Veterinary Medicine Cluj-Napoca, Cluj-Napoca, Romania; 2 Department of Surgery and ICU, Faculty of Veterinary Medicine, University of Agricultural Sciences and Veterinary Medicine Cluj-Napoca, Cluj-Napoca, Romania; 3 Department of Veterinary Pathology, Faculty of Veterinary Medicine, University of Agricultural Sciences and Veterinary Medicine Cluj-Napoca, Cluj-Napoca, Romania; 4 Department of Imaging, Faculty of Veterinary Medicine, University of Agricultural Sciences and Veterinary Medicine Cluj-Napoca, Cluj-Napoca, Romania; 5 Department of Pathophysiology, Faculty of Veterinary Medicine, University of Agricultural Sciences and Veterinary Medicine Cluj-Napoca, Cluj-Napoca, Romania

**Keywords:** abdominal wall reconstruction, exotic animal surgery, fascia lata autograft, inguinal hernia, rabbit surgery

## Abstract

**Introduction:**

Inguinal hernias are uncommon but significant pathologies in rabbits, particularly in geriatric patients, in whom comorbidities, tissue fragility, and anesthetic risk complicate surgical management.

**Methods:**

An 8-year-old, 850 g male Netherland Dwarf rabbit presented with a right inguinal hernia, reducible on palpation. Computed tomography and ultrasonography confirmed herniation of the cecum and urinary bladder. Initial surgical management under general anesthesia included orchidectomy, cystopexy, and primary closure of the inguinal canal using atraumatic monofilament polydioxanone (PDO) 4–0 suture. One month postoperatively, recurrence of the hernia involving the cecum was observed. A second reconstructive procedure was therefore performed using a fascia lata autograft collected from the patient’s right pelvic limb. After repositioning the herniated organs, the graft was sutured with atraumatic monofilament PDO 4–0.

**Results:**

Clinical and ultrasonographic evaluation at 10 days postoperatively demonstrated continuity of the reconstructed abdominal wall and absence of recurrent herniation, while computed tomography at three months and ultrasonography at six months showed maintenance of the repair without recurrence.

**Conclusion:**

To the authors’ knowledge, this represents the first reported use of a fascia lata autograft for recurrent inguinal hernia repair in a pet rabbit. This case suggests that fascia lata autografting may represent a feasible reconstructive option in selected rabbits when primary suture closure is considered insufficient. Further studies are required to evaluate long-term outcomes and recurrence rates in exotic companion mammals.

## 1 Introduction

Hernias in domestic rabbits (*Oryctolagus cuniculus*) are relatively uncommon pathologies that can carry a severe prognosis. They occur when abdominal or pelvic organs protrude through defects in the abdominal wall, diaphragm, pelvis, inguinal canals, or other supporting structures. Their clinical significance is amplified by the small size of rabbits, their fragile soft tissues, and unique anatomical features, which complicate both diagnosis and surgical management. In dwarf breed rabbits, the relatively delicate soft tissues and limited dimensions of the inguinal region may further reduce the operative space available for manipulation and reconstruction, increasing the technical difficulty of surgical repair. In geriatric individuals, age-related tissue fragility, reduced body condition, and concurrent disease may additionally compromise tissue strength, wound healing, and the durability of primary closure. Geriatric and systemically compromised rabbits may present additional perioperative challenges, emphasizing the need for thorough preoperative assessment, careful surgical planning, and meticulous intraoperative technique (1, 2). Complex inguinal hernias in rabbits may require detailed diagnostic evaluation and individualized surgical planning, particularly because of the anatomical characteristics of the inguinal region and the potential for recurrence (3).

Inguinal hernias occur through the inguinal canal and may involve organs such as the omentum, intestinal loops, or the urinary bladder. These cases are surgically challenging due to the limited operative space and fragile tissues, and recurrence is common in elderly or cachectic rabbits (3, 4). Hernias involving intestinal loops carry a high risk of intestinal obstruction, while those involving the urinary bladder increase the likelihood of urinary obstruction, infection, or chronic inflammation (4–8). More complex hernias involving multiple organs present additional surgical challenges and often require detailed imaging to guide preoperative planning (3, 7).

Published clinical reports indicate that hernias in domestic rabbits may occur at several anatomical locations and may involve different abdominal or pelvic structures. Selected naturally occurring hernias reported in domestic rabbits are summarized in Table 1. Identifying the exact location of the defect helps determine the surgical approach, while understanding which organs are involved guides perioperative management and helps anticipate complications such as obstruction, infection, or recurrence.

**Table 1:** Selected published clinical reports of naturally occurring hernias in domestic rabbits (*Oryctolagus cuniculus*).

Hernia type/location	Reported herniated structure(s)	References
Inguinal/inguinal-scrotal	Urinary bladder	(3, 4, 6, 30)
Perineal	Urinary bladder	(31)
Umbilical	Intestines and fat	(32)
Diaphragmatic/retroperitoneal	Kidney and perirenal fat	(7)

When primary closure is compromised by poor tissue quality or recurrent defects, reinforcement with an additional reconstructive material may be required. Fascia lata offers several potential advantages in this context: as an autologous tissue, it provides mechanical reinforcement while avoiding implantation of permanent synthetic material and the associated concerns regarding foreign-body reaction, infection, or adhesion formation. Its biological compatibility, tensile properties, and capacity for incorporation into surrounding tissues make it a potentially useful reconstructive option when tissue fragility or previous surgical failure limits conventional closure (8–10).

The present case report describes the surgical management of a recurrent inguinal hernia in a geriatric Netherland Dwarf breed pet rabbit, a patient in which the limited dimensions of the inguinal region, reduced operative space, age-related tissue fragility, poor body condition, and concurrent disease increased the technical and biological challenges of repair. Following failure of primary closure, reconstruction was performed using a fascia lata autograft, as it provides autologous, mechanically supportive tissue capable of reinforcing the defect without introducing permanent synthetic material, an important consideration in a rabbit patient with compromised local tissue quality and a recurrent defect. The aim of this report is to detail the diagnostic work-up, surgical technique, and postoperative follow-up, and to highlight the feasibility of autologous fascia lata as a reconstructive option for recurrent abdominal wall defects in tissue-compromised exotic companion mammals.

## 2 Case presentation

An 8-year-old, 850 g male Netherland Dwarf rabbit was presented to the New Companion Animals Clinic, Faculty of Veterinary Medicine, University of Agricultural Sciences and Veterinary Medicine Cluj-Napoca (Romania), for evaluation of a right inguinal swelling, described as intermittent and reducible upon palpation. The patient’s medical history was notable for chronic dental malocclusion managed with periodic dental trimming. On physical examination, the rabbit had a body condition score of 2/5 (lean), assessed using the five-point Rabbit Size-O-Meter body condition scoring system, based on visual assessment and palpation of anatomical landmarks including the ribs, spine, and pelvis. A score of 2/5 is consistent with a lean body condition, characterized by reduced soft-tissue coverage and easily palpable bony prominences. The patient’s medical history included chronic dental malocclusion and chronic rhinitis, both of which were confirmed by computed tomography of the head. The dental abnormalities were considered responsible for impaired prehension and reduced feeding efficiency, while the concurrent chronic rhinitis may have further contributed to decreased food intake. The owner reported a decreased appetite and the production of small fecal pellets. The reduced fecal pellet size was considered compatible with decreased food intake and possible gastrointestinal hypomotility rather than being attributed solely to reduced nutritional intake. On physical examination, the patient was bright, alert, reactive, with normal hydration, and heart rate (HR) was 250 beats/min, respiratory rate (RR) was 180 breaths/min, and rectal temperature was 38.6 °C. Palpation of the inguinal region identified a soft, reducible protrusion through the right inguinal canal, while the contralateral side was unremarkable. No pain response or discomfort was observed during abdominal or inguinal palpation or during gentle reduction of the hernia. At presentation, the rabbit remained active but had slightly decreased appetite and reduced food intake.

Diagnostic imaging was performed in two stages. Ultrasonography was first performed under mild sedation with intramuscular (IM) midazolam (0.5 mg/kg) to assess the extent and contents of the hernia. Ultrasonography suggested herniation of both the urinary bladder and cecum through the right inguinal canal. Computed tomography (CT) was performed the following day for more precise anatomical localization and surgical planning, under deep sedation using IM midazolam (0.5 mg/kg), medetomidine (0.05 mg/kg) and ketamine (3 mg/kg). Oxygen 100% was delivered via a facial mask throughout the procedure, and an intravenous (IV) catheter was placed in the marginal right auricular vein. CT was performed using a SOMATOM Scope scanner (Siemens Healthineers), with the rabbit positioned in sternal recumbency. Intravenous iodinated contrast medium (iohexol, Omnipaque™ 350 mg I/mL, GE Healthcare) was administered at 1 mL/kg without dilution. Images were reconstructed using a soft-tissue algorithm and evaluated using multiplanar reconstructions. Physiological monitoring included peripheral oxygen saturation (SpO₂) using a multiparameter monitor (Mindray PM9000 Vet, Shenzhen, China), while HR was assessed using an EICKEMEYER® Ultrasonic Doppler (Eickemeyer – Medizintechnik für Tierärzte KG, Tuttlingen, Germany) probe positioned over the cardiac region. During CT, sudden bradycardia was observed, with a minimum heart rate of approximately 120 beats/min, together with dyspnea. Atropine (0.1 mg/kg IV) was administered, and oxygen supplementation via facial mask was continued. Furthermore, atipamezole was administered IM at 0.2 mg/kg, and the patient was monitored until cardiorespiratory stability was achieved. CT confirmed herniation of a cecal loop and the urinary bladder through the right inguinal canal, without evidence of incarceration or strangulation (Figures 1A,C). Contrast enhancement and subsequent urinary excretion facilitated delineation of the urinary bladder and its displacement through the inguinal canal. The hernial defect measured approximately 1.5 × 1 cm on CT imaging. No definitive underlying cause was identified; however, the rabbit’s low body condition, reduced food intake, and geriatric status were considered possible contributing factors.

**Figure 1:**
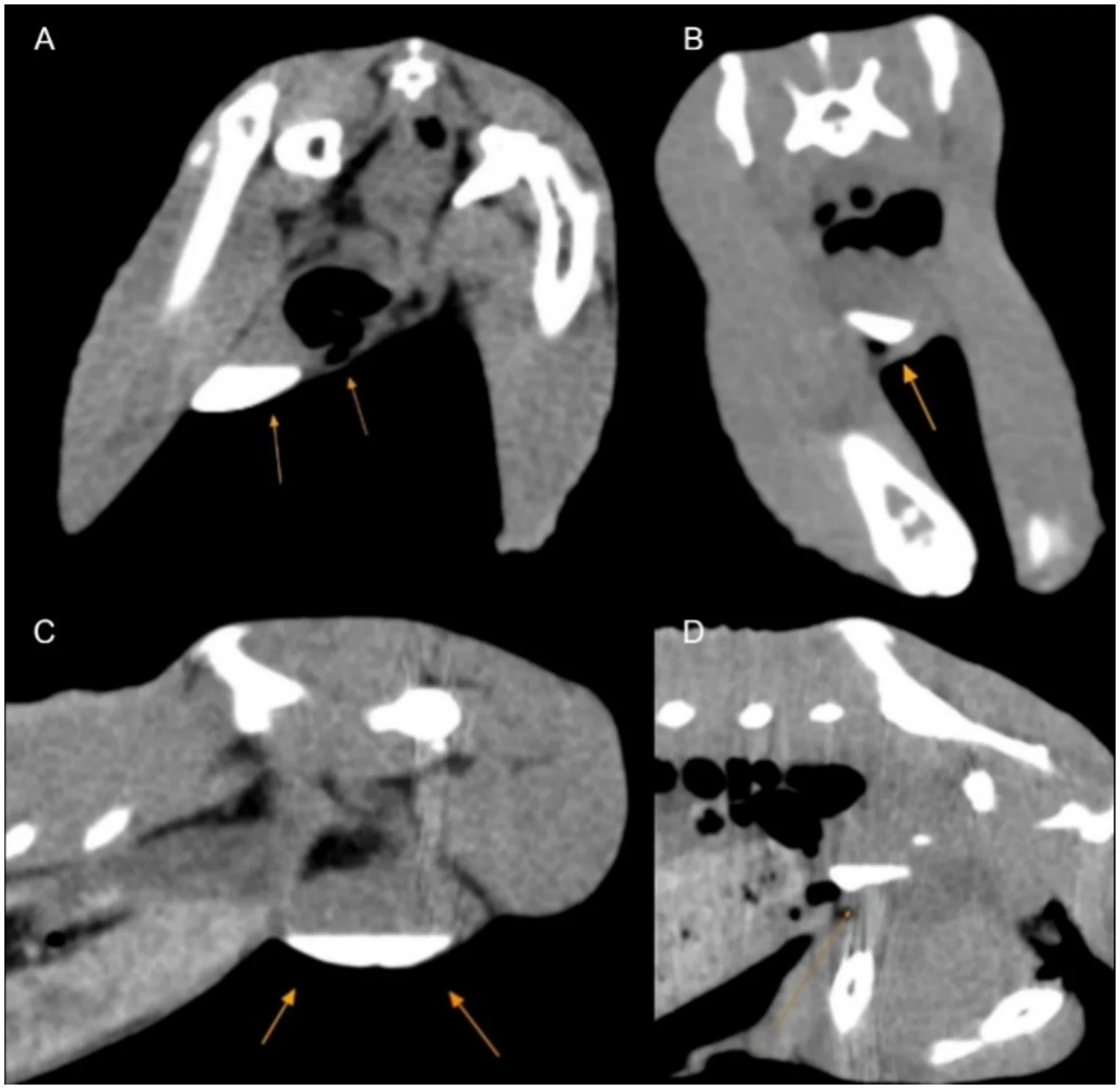
Preoperative and postoperative CT evaluation of the recurrent inguinal hernia: **(A)** Preoperative axial CT images demonstrate herniation of the cecum and urinary bladder (arrows) into the right inguinal region. Contrast opacification of the urinary bladder following intravenous administration of iohexol delineates its displacement through the right inguinal canal. The hernial defect measures approximately 1.5 × 1 cm, with no evidence of incarceration or strangulation. **(B)** Postoperative axial CT images obtained 3 months after surgery show continuity of the reconstructed right inguinal region, with no evidence of recurrent herniation, graft displacement, or abnormal organ displacement. The fascia lata autograft is visible at the repair site (arrow). **(C)** Preoperative sagittal CT images further illustrate displacement of the urinary bladder (arrows) through the right inguinal canal. **(D)** Postoperative sagittal CT images demonstrate maintenance of abdominal wall continuity, with no evidence of recurrent herniation, graft displacement, or abnormal organ displacement.

Complete blood count (CBC) and serum biochemical analyses were performed at presentation. The CBC was carried out on a BC-60 R hematology analyzer (Mindray) and blood smears were stained with a rapid May-Grunwald-Giemsa (Optica, Italy). The CBC revealed lymphopenia (0.28 × 10^9^/L; RI: 1.25–6.35 × 10^9^/L) accompanied by an increased heterophil-to-lymphocyte (H: L) ratio of 10.6:1. Serum biochemical analysis was performed using the VETSCAN Comprehensive Diagnostic Profile Rotor with the Zoetis VETSCAN VS2 Chemistry Analyzer. Reference intervals for rabbit biochemical parameters were obtained from Carpenter’s Exotic Animal Formulary, 6th ed. (11). Preoperative serum biochemical analysis revealed several abnormalities compared with established reference intervals for rabbits. A markedly decreased albumin concentration (<1.0 g/dL; RI: 2.8–4.0 g/dL) was identified in a markedly hemolyzed sample (HEM 3+). Alkaline phosphatase activity was increased (95 U/L; RI: 6–14 U/L), whereas alanine aminotransferase activity was slightly below the stated reference interval (51 U/L; RI: 52–80 U/L). Total serum calcium was increased (14.9 mg/dL; RI: 7.6–12.2 mg/dL), and potassium was mildly increased (5.6 mEq/L; RI: 3.4–5.1 mEq/L). Blood urea nitrogen was within the reference interval (13 mg/dL; RI: 9–29 mg/dL), whereas creatinine was below the stated reference interval (0.6 mg/dL; RI: 1.0–2.2 mg/dL). Glucose was increased (229 mg/dL; RI: 109–161 mg/dL). Other measured biochemical parameters were within their respective reference intervals.

Initial surgical management was performed under general anesthesia using a balanced multimodal protocol consisting of midazolam (0.8 mg/kg), methadone (0.5 mg/kg), medetomidine (0.03 mg/kg), and ketamine (3 mg/kg). This anesthetic protocol was selected because the procedure involved multiple highly nociceptive components, including urinary bladder manipulation and cystopexy, abdominal wall and inguinal canal suturing, and orchidectomy, requiring reliable anesthesia and multimodal analgesia despite the patient’s geriatric status and low body condition. Following catheterization of the left saphenous vein, the patient was induced with propofol 2 mg/kg IV. To facilitate endotracheal intubation, lidocaine 2% (1 mg/kg) was applied intranasally. This route was intentionally selected because rabbits are obligate nasal breathers, allowing the local anesthetic to pass through the nasopharynx toward the pharyngeal and epiglottic region before airway instrumentation. Intubation was performed under direct visualization using a rigid endoscope (1.9 mm, Eickemeyer, Germany), and a 2,0 mm ID uncuffed endotracheal tube was advanced into the trachea. After securing the airway, the rabbit was connected to an Ayre’s T-piece non-rebreathing circuit, and anesthesia was maintained with 1–1.5% isoflurane in 100% oxygen. Intraoperative monitoring was performed using a Henry Schein Veterinary Patient Monitor HS-VPM-15 (Henry Schein, Inc., Melville, NY, USA) and included SpO₂, electrocardiography (ECG), end-tidal CO₂, HR, RR, and rectal body temperature. Rectal temperature was monitored throughout anesthesia. Heart rate was additionally assessed using Ultrasonic Doppler equipped with an 8-MHz probe. After induction, a continuous infusion of lidocaine (40 μg/kg/min) and ketamine (10 μg/kg/min) was initiated to provide multimodal intraoperative analgesia and reduce inhalant anesthetic requirements. Two additional ketamine boluses (2 mg/kg) were administered when HR and RR increased by 20% from baseline values, indicating inadequate nociceptive control during surgical stimulation. External thermal support was provided using a heating pad throughout the procedure.

Orchidectomy was performed during the first procedure. Following castration, primary closure of the inguinal canal was performed using atraumatic monofilament PDO 4–0 sutures (AssuCryl® MonoSlow, Assut Sutures, Switzerland). To prevent recurrence of urinary bladder herniation, a cystopexy was performed, securing the bladder to the right abdominal wall using a single atraumatic monofilament PDO 5–0 suture (AssuCryl® MonoSlow, Assut Sutures, Switzerland) placed in the scarified abdominal wall to provide stable fixation. Immediate postoperative management included analgesia with a lidocaine continuous-rate infusion (100 μg/kg/min IV; solution concentration 0.6 mg/mL, administered at 10 mL/kg/h for 24 h), meloxicam (1 mg/kg q24h SC/PO for 7 days), and buprenorphine (0.05 mg/kg IV/SC q6–8h for 72 h). Antibiotic therapy consisted of trimethoprim–sulfonamide (30 mg/kg q12h PO for 10 days). The patient recovered uneventfully, with normal ambulation and appetite.

However, one month postoperatively, recurrence of cecal herniation was observed. The urinary bladder had not re-herniated at the time of recurrence. Given the recurrent defect and the reduced strength of the abdominal wall tissues, reconstructive repair using a fascia lata autograft was elected. No repeat hematological or biochemical analyses were performed immediately before the second surgery because the owner declined additional laboratory testing for financial reasons. The patient underwent a second general anesthesia using the same protocol applied during the initial surgical procedure. The anesthetic protocol was maintained because the second intervention was expected to be prolonged and markedly nociceptive, involving re-exploration of the inguinal region, manipulation and closure of the abdominal wall defect, harvesting of the fascia lata graft, and fixation of the graft to the abdominal wall. However, this surgical protocol had been adjusted relative to the injectable protocol used for CT scan by reducing the medetomidine dose from 0.05 to 0.03 mg/kg and increasing the midazolam dose from 0.5 to 0.8 mg/kg.

A 1.5 × 2 cm fascia lata graft was harvested from the lateral aspect of the right pelvic limb (Figures 2A,B). Following a small craniolateral skin incision, the subcutaneous tissue was bluntly dissected to expose the fascia lata. The anatomical margins were defined proximally by the tensor fasciae latae muscle, cranially by the sartorius muscle, caudally by the cranial border of the biceps femoris muscle, and distally by the distal third of the femur toward the lateral femoral condyle and stifle region. Given the small body size and relatively thin musculofascial layers of the rabbit hindlimb, dissection was performed carefully to avoid injury to the underlying musculature and neurovascular structures. The fascia lata was elevated and excised as a rectangular graft, then placed on sterile gauze moistened with 0.9% saline until implantation. After saline lavage, the donor site was closed in layers using 4–0 monofilament polydioxanone suture.

**Figure 2:**
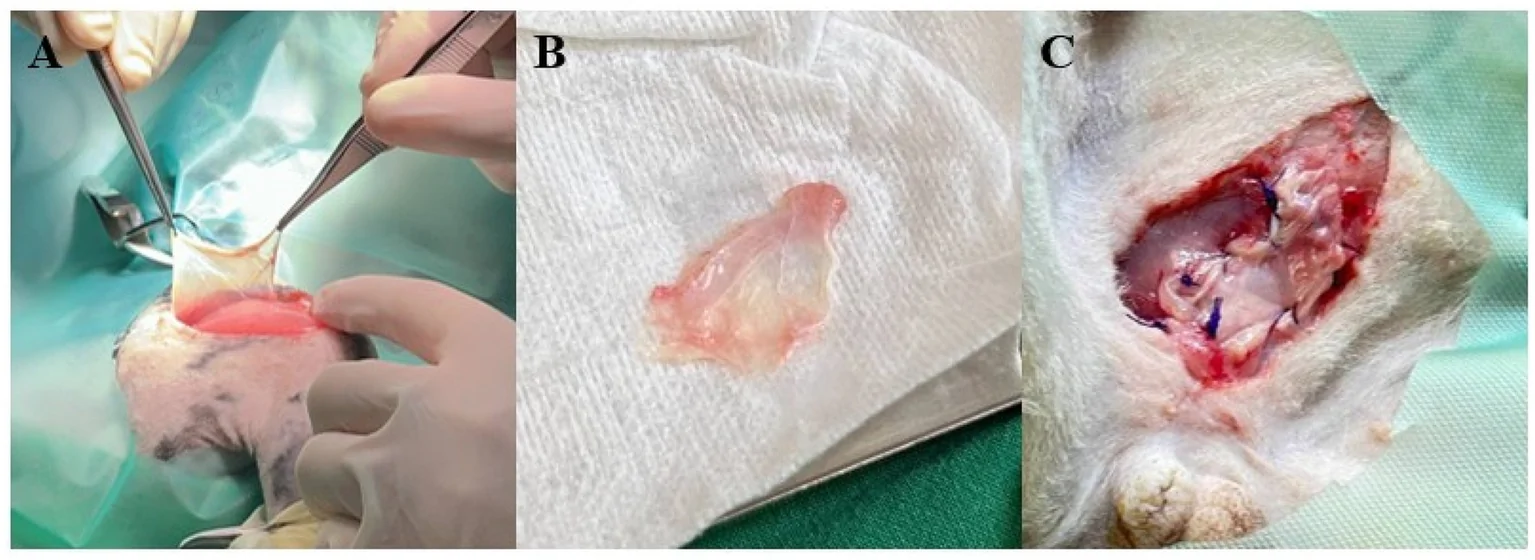
Fascia lata autograft harvesting and surgical repair of recurrent inguinal hernia **(A)** Intraoperative images showing harvesting of a fascia lata graft from the right pelvic limb, with careful dissection to preserve surrounding musculature and minimize donor-site trauma. **(B)** The harvested fascia lata graft (approximately 1.5 × 2 cm) prepared for implantation. **(C)** Intraoperative view of the recurrent inguinal hernia following cecum repositioning. The fascia lata autograft is sutured over the defect using an atraumatic 4–0 monofilament polydioxanone (PDO) suture (AssuCryl® MonoSlow, Assut Sutures, Switzerland), achieving tension-free closure and reinforcement of the inguinal canal.

During the second surgery, the hernial sac was opened and carefully explored. Mild adhesions between the cecum and surrounding tissues were gently dissected, and the cecum was repositioned into the abdominal cavity. The 1.5 × 2 cm fascia lata graft was positioned over the external surface of the inguinal defect, extending approximately 3 mm beyond the defect margins. It was secured circumferentially to the surrounding abdominal wall using interrupted cruciate sutures with 4–0 monofilament polydioxanone (PDO) on an atraumatic round-bodied needle (AssuCryl® MonoSlow, Assut Sutures, Switzerland) (Figure 2C). The graft was apposed without undue tension. Standard closure of the surgical wound was performed in two layers. Postoperative management was identical to that used after the initial surgical procedure, including lidocaine CRI, meloxicam, buprenorphine, trimethoprim–sulfonamide, and supportive care with monitoring of wound healing and recurrence. The patient resumed voluntary food intake approximately 4 h postoperatively and was monitored for postoperative pain using the Rabbit Grimace Scale (12).

## 3 Results

Histological examination of the testes confirmed diffuse, non-neoplastic testicular atrophy. Follow-up included clinical evaluation and ultrasonography at 10 days postoperatively. At that time, the surgical wounds were healed, no recurrent herniation was detected clinically, and ultrasonography demonstrated continuity of the reconstructed abdominal wall without evidence of fluid accumulation or displacement of the graft. Because histopathological evaluation of the graft was not performed, complete histological integration could not be confirmed at this stage. CT imaging performed at three months confirmed absence of recurrence (Figures 1B,D).

At six months postoperatively, ultrasonographic evaluation of the inguinal region showed no evidence of recurrent herniation or other abnormalities associated with the repair (Figure 3). The rabbit maintained normal appetite, activity, and urination, and no clinically apparent complications related to graft harvest or implantation were observed during the six-month follow-up period.

**Figure 3:**
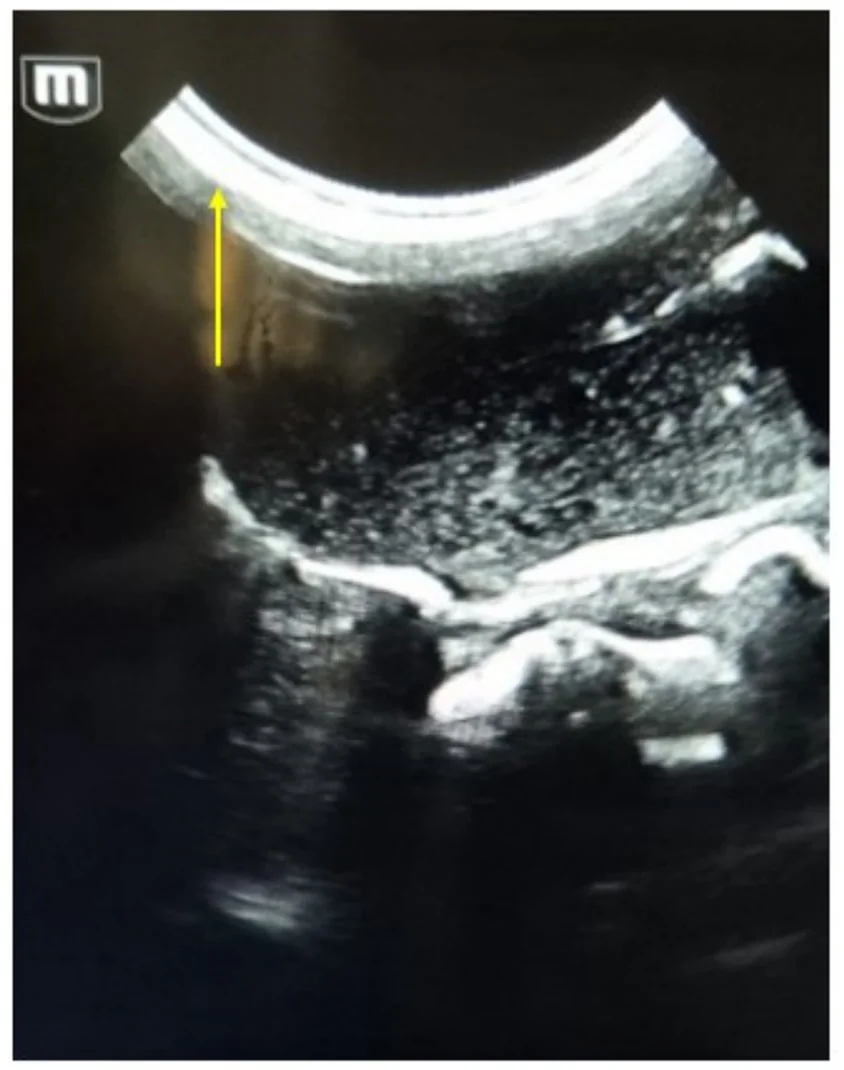
Transverse ultrasonographic view of the repaired right inguinal region at six months postoperatively, showing continuity of the reconstructed abdominal wall without evidence of recurrent herniation, fluid accumulation, or graft displacement. The graft remains identifiable at the repair site (arrow), with preserved continuity of the adjacent abdominal wall layers.

Hematological and serum biochemical analyses were repeated at the six-month recheck, and the results at presentation and at six months are summarized in Table 2. Blood smear examination revealed a moderate number of reactive lymphocytes with plasmacytoid morphology and H: L ratio of 2.9:1. Serum biochemistry showed persistent mild hypoalbuminemia (2.4 g/dL; RI: 2.8–4.0 g/dL) and increased globulin concentration (4.9 g/dL; RI: 2.1–3.7 g/dL). Alkaline phosphatase activity remained increased at 44 U/L (RI: 6–14 U/L), whereas alanine aminotransferase activity was within the reference interval. Serum calcium remained increased at 15.7 mg/dL (RI: 7.6–12.2 mg/dL), and potassium was mildly increased at 5.9 mEq/L (RI: 3.4–5.1 mEq/L). Blood urea nitrogen was 15 mg/dL (RI: 9–29 mg/dL) and creatinine was 1.2 mg/dL (RI: 1.0–2.2 mg/dL). At the time of evaluation, purulent nasal discharge associated with chronic rhinitis and dental disease was also present.

**Table 2:** Hematological and serum biochemical findings at presentation and at the six-month follow-up.

Parameter	Presentation	Six-month follow-up	Reference interval
Hematology			
WBC (×10^9^/L)	4.60	7.67	3.00–12.50
Heterophils/neutrophils (×10^9^/L)	3.77	5.50	1.02–5.95
Lymphocytes (×10^9^/L)	0.28	0.97	1.25–6.35
Monocytes (×10^9^/L)	0.35	0.79	0.00–1.85
Eosinophils (×10^9^/L)	0.01	0.07	0.00–0.18
Basophils (×10^9^/L)	0.19	0.34	0.00–0.65
RBC (×10^12^/L)	5.69	6.20	4.15–6.50
Hemoglobin (g/L)	131	129	85–165
Hematocrit (%)	38.4	39.9	25.0–42.0
MCV (fL)	67.5	64.4	60.0–80.0
MCH (pg)	23.1	20.8	17.0–24.0
MCHC (g/L)	341	323	270–340
Platelets (×10^9^/L)	136*	397	200–800
Heterophil:lymphocyte ratio (calculated from the manual blood-smear differential)	10.6:1	2.9:1	—
Blood smear	Mature heterophils 85%, lymphocytes 8%, monocytes 6%, eosinophils 1%, basophils 0%; moderate platelet aggregates	Immature heterophils 2%, mature heterophils 66%, lymphocytes 23%, monocytes 5.5%, eosinophils 0.5%, basophils 3%; moderate reactive plasmacytoid lymphocytes	—
Serum biochemistry			
Albumin (g/dL)	<1.0	2.4	2.8–4.0
Alkaline phosphatase, ALP (U/L)	95	44	6–14
Alanine aminotransferase, ALT (U/L)	51	65	52–80
Amylase (U/L)	209	149	82–343
Total bilirubin (mg/dL)	0.2	0.3	0.1–0.5
Blood urea nitrogen, BUN (mg/dL)	13	15	9–29
Calcium (mg/dL)	14.9	15.7	7.6–12.2
Phosphorus (mg/dL)	5.4	5.0	3.0–6.2
Creatinine (mg/dL)	0.6	1.2	1.0–2.2
Glucose (mg/dL)	229	178	109–161
Sodium (mmol/L)	143	141	138–148
Potassium (mmol/L)	5.6	5.9	3.4–5.1
Total protein (g/dL)	6.9	7.4	6.1–7.5
Globulin (g/dL)	>5.9	4.9	2.1–3.7

Serum biochemical analyses were performed using the VETSCAN Comprehensive Diagnostic Profile Rotor with the Zoetis VETSCAN VS2 Chemistry Analyzer. Reference intervals for rabbit biochemical parameters were obtained from Carpenter’s Exotic Animal Formulary, 6th ed. (11). The presentation sample showed marked hemolysis (HEM 3+), whereas the six-month sample showed no hemolysis, lipemia, or icterus (HEM 0, LIP 0, ICT 0). *Moderate platelet aggregates were observed on blood-smear examination.

A chronological summary of diagnostic investigations, surgical interventions, recurrence, and follow-up is presented in Scheme 1.

**SCHEME 1:**
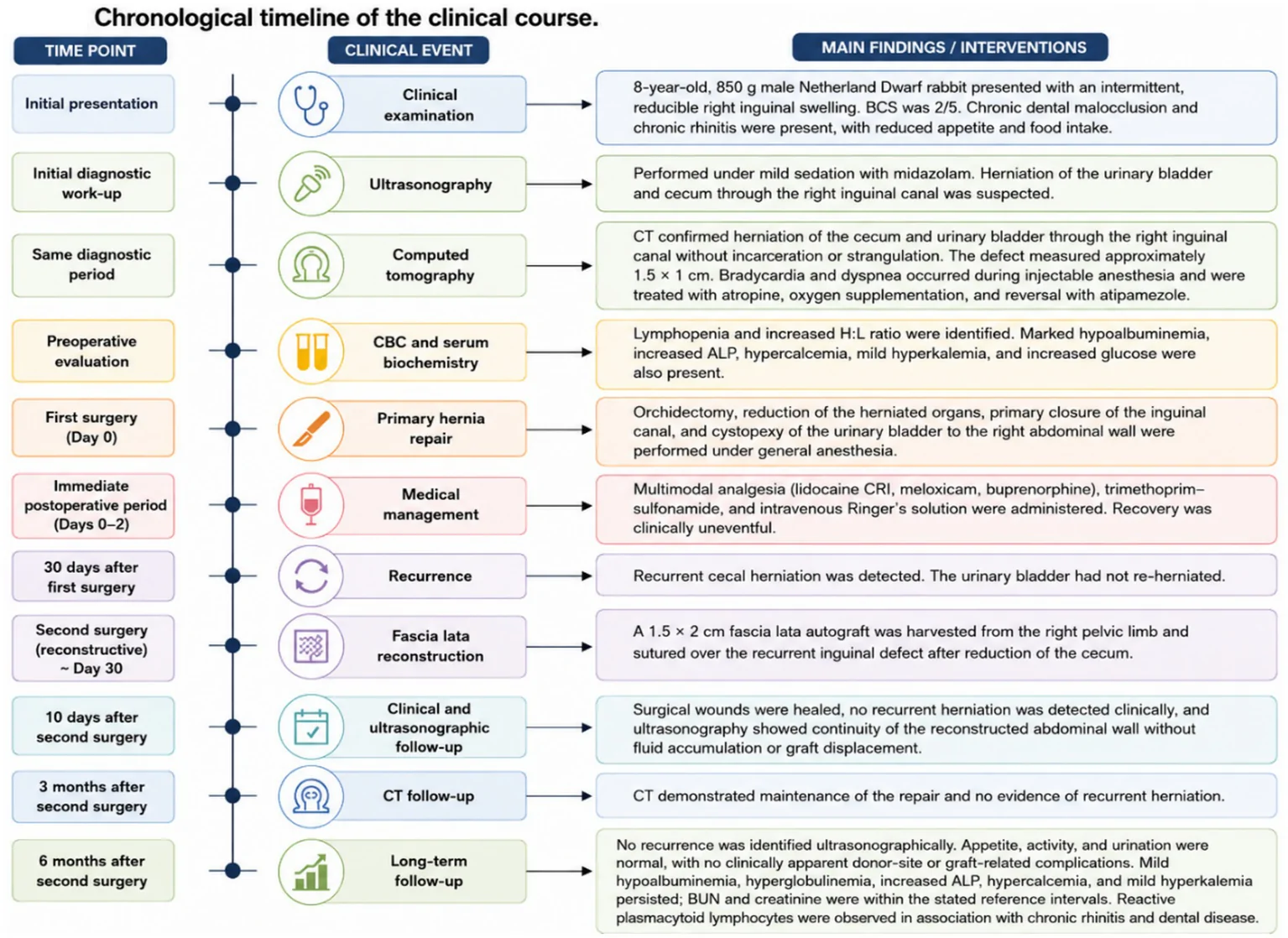
Case timeline. Chrological tiemline of the clinical course. The visual design of the scheme was developed with assistance from OpenAI, based exclusively on clinical information provided and verified by the authors.

## 4 Discussion

Recurrent inguinal hernia repair in this rabbit was complicated by several patient-specific factors, including advanced age, low body condition, marked hypoalbuminemia, previous surgical manipulation, and limited tissue available for reconstruction. These features reduced confidence in a second primary closure and supported the use of an autologous reinforcing material. Interest in synthetic and composite materials for abdominal wall reconstruction has increased because of their potential to provide mechanical reinforcement in complex or recurrent defects (13). Although synthetic and composite meshes have demonstrated favorable biomechanical properties and tissue incorporation in experimental rabbit models, their clinical performance in geriatric, tissue-fragile pet rabbits remain poorly documented (14–18). Experimental studies have shown that different biomaterials may produce distinct patterns of tissue integration, collagen deposition, and inflammatory response (14), while synthetic and composite meshes may improve tensile strength and abdominal wall stability compared with simple closure techniques (16). In the present case, fascia lata was selected because it provided autologous tissue with mechanical strength while avoiding permanent synthetic material in a previously operated inguinal region. This consideration was particularly relevant because permanent implants may be associated with foreign-body reaction, adhesion formation, infection, or other implant-related complications, although such risks depend on the material and surgical environment (13).

Experimental rabbit models have also contributed substantially to the evaluation of reconstructive strategies for abdominal wall defects. Models involving large or chronic abdominal wall hernias have demonstrated the usefulness of this species for investigating long-term tissue remodeling and biomechanical behavior (17, 18). Three-dimensional polypropylene and composite meshes have demonstrated the ability to provide structural support while permitting tissue ingrowth in experimental rabbit models (15, 19). Other minimally invasive approaches, including laparoscopic tissue-adhesive techniques for inguinal hernia repair, have also been investigated experimentally in rabbits (5). Nevertheless, extrapolation of these experimental findings to an elderly, low-body-condition companion rabbit should be made cautiously.

Fascia lata has an established role as an autologous reconstructive material in veterinary and human surgery. In dogs, fascia lata grafts have been used for reinforcement of perineal and abdominal wall defects, providing mechanical support in situations where local tissues are compromised (9, 20). Clinical use of autologous fascia lata flaps in dogs has also been reported for perineal hernia repair, supporting its clinical feasibility as an autologous reconstructive material (21). In human reconstructive surgery, fascia lata has likewise been used as an autologous reinforcement material, including in complex or recurrent abdominal wall defects, because it can provide durable mechanical support without implantation of permanent synthetic material (22).

Experimental studies in rabbits have also demonstrated the feasibility of fascia lata for urinary tract reconstruction, including urethral and urinary bladder repair (8, 10). Patch urethroplasty using autologous fascia lata has been associated with satisfactory tissue incorporation and limited postoperative fibrosis (10), while experimental urinary bladder reconstruction has shown favorable postoperative evolution (8). Histological evaluation of human cadaveric fascia lata implanted in a rabbit model demonstrated progressive host-tissue incorporation, neovascularization, and replacement of graft collagen by host collagen over time (23). These reports support the biological compatibility of fascia lata in rabbits, although they concern different anatomical sites and experimental indications. Therefore, the relevance of the present case lies not in the novelty of fascia lata as a graft material itself, but in its clinical use for recurrent inguinal hernia repair in a geriatric companion rabbit after failure of primary closure.

The recurrence observed 30 days after the first surgery may have been influenced by the patient’s compromised nutritional and tissue condition. At presentation, the rabbit had a BCS of 2/5 and marked hypoalbuminemia (<1 g/dL), together with reduced food intake. These abnormalities may indicate inadequate protein and nutritional status and are clinically relevant because protein availability is important for fibroblast activity, collagen synthesis, wound maturation, and maintenance of tissue strength. Consequently, the combination of severe hypoalbuminemia and low body condition may have reduced the mechanical quality of the abdominal wall and impaired healing of the primary repair. Previous surgical manipulation and poor tissue quality may also have reduced confidence in repeat primary closure in this patient. However, because this is a single clinical case, a direct causal relationship between these abnormalities and recurrence cannot be established.

In addition to poor body condition and hypoalbuminemia, the patient had chronic dental malocclusion and chronic rhinitis, both confirmed by computed tomography, and purulent nasal discharge was present at the six-month evaluation. These concurrent conditions may have contributed indirectly to impaired healing through reduced food intake and persistent inflammatory stimulation. Consistent with these systemic concerns, hematological evaluation at presentation revealed lymphopenia and an increased heterophil-to-lymphocyte ratio, findings compatible with physiological stress and potentially influenced by the patient’s chronic dental and respiratory disease. On biochemistry, the marked hypoalbuminemia was considered particularly relevant because, together with the low body condition and reduced food intake, it may have reflected compromised nutritional status and reduced capacity for tissue repair, thereby contributing to reduced strength of the primary closure. Although the sample was markedly hemolyzed, the severity of hypoalbuminemia was not attributed solely to hemolysis. Hypercalcemia was interpreted cautiously because of the concurrent severe hypoalbuminemia and was not ascribed solely to the characteristic calcium metabolism of rabbits. Mild hyperkalemia may have been influenced by hemolysis, although altered urinary outflow associated with urinary bladder herniation could not be excluded as a contributing factor. The increased glucose concentration was considered compatible with a stress-related response. Overall, these abnormalities were interpreted in the context of the patient’s geriatric status, chronic dental and respiratory disease, reduced food intake, poor body condition, and peri-diagnostic stress.

At the six-month recheck, some of these systemic abnormalities persisted. The H: L ratio had decreased from 10.6:1 at presentation to 2.9:1. Reactive lymphocytes with plasmacytoid morphology and increased globulin concentration were identified, findings compatible with ongoing antigenic or inflammatory stimulation. At the time of evaluation, the rabbit had purulent nasal discharge associated with chronic rhinitis and dental disease, further supporting persistent inflammatory stimulation. On biochemistry, the persistence of mild hypoalbuminemia at this time may also have reflected the combined effects of chronic inflammatory disease and nutritional compromise, although a specific causal mechanism could not be established. In the absence of clinical or imaging evidence of complications related to the hernia repair, these abnormalities were considered more consistent with the concurrent chronic upper respiratory and dental disease than with the surgical procedure. Nevertheless, chronic inflammation, reduced nutritional intake, poor body condition, and biochemical evidence of nutritional compromise represent additional systemic factors that may influence tissue repair and should be considered when planning reconstructive surgery in geriatric rabbits.

Regarding the persistent hypercalcemia, dietary calcium intake was also considered. The rabbit was fed Timothy hay and a commercial adult-rabbit pellet diet. Rabbits have a distinctive calcium metabolism characterized by relatively efficient intestinal calcium absorption and an important role of urinary excretion in calcium homeostasis (11). Therefore, dietary calcium intake may influence serum calcium concentrations. However, in the present case, the increased total calcium concentration was interpreted cautiously and was not attributed solely to species-specific calcium metabolism or diet, particularly given the concurrent marked hypoalbuminemia at presentation and the persistence of hypercalcemia at follow-up.

Diffuse, non-neoplastic testicular atrophy was identified histologically. Although its direct role in the development of the inguinal hernia cannot be established, age-related testicular atrophy may have contributed to changes in the local inguinal anatomy. Together with the patient’s low body condition and reduced tissue strength, this may have facilitated enlargement of the available space within the inguinal region and predisposed to herniation. This interpretation remains speculative and should be considered cautiously.

Donor-site morbidity is an important consideration when harvesting fascia lata in a small patient. In this 850 g rabbit, a 1.5 × 2 cm graft represented a relatively substantial harvest in relation to body size. Therefore, postoperative assessment included observation of ambulation, hind-limb use, wound healing, and local abnormalities at the donor site. No limping, gait disturbance, functional impairment of the right pelvic limb, wound complication, or clinically apparent muscle herniation was observed at any follow-up examination during the six-month postoperative period. Nevertheless, fascia lata harvesting involves additional surgical time and carries the potential for postoperative discomfort and donor-site morbidity; transient postoperative lameness has been reported following fascia lata harvesting in dogs (20, 21). Although graft harvesting appeared to be well tolerated in this individual, more subtle alterations in limb biomechanics were not objectively quantified, and this remains a limitation.

The cystopexy performed during the first surgery was intended to provide temporary stabilization of the urinary bladder against the scarified right abdominal wall and to promote adhesion formation rather than to function as the sole long-term mechanical support of the repair. Cystopexy has recently been described in a rabbit with traumatic inguinoscrotal bladder exteriorization, in which the urinary bladder was fixed to the right internal abdominal wall musculature using simple interrupted 4–0 absorbable monofilament sutures following gentle seromuscular scarification (24). In the present case, fixation with a single PDO 5–0 suture represents a potential biomechanical limitation because the load is concentrated at one fixation point and may provide less resistance to displacement than a broader or multi-point fixation pattern. Although the urinary bladder did not re-herniate at the time of recurrence, the mechanical strength of the cystopexy was not objectively evaluated. Therefore, the contribution of the single-suture fixation to long-term bladder stabilization cannot be determined from this case, and multi-point fixation or a broader appositional technique may provide greater mechanical security in future cases.

Perioperative management represented an important challenge in this case because the rabbit was 8 years old, had a low body condition score, and had previously developed bradycardia and dyspnea during CT examination under an injectable anesthetic protocol. Rabbits require careful perioperative management because of their species-specific physiological characteristics and susceptibility to anesthetic complications, particularly in compromised patients (2, 25). The surgical procedures were markedly nociceptive and included urinary bladder manipulation and cystopexy, orchidectomy, abdominal wall reconstruction, and, during the second intervention, fascia lata harvesting and graft fixation. Multimodal perioperative analgesia is therefore particularly important in rabbits, in which pain assessment may be challenging and a single analgesic technique may be insufficient for highly nociceptive procedures (26). A balanced multimodal anesthetic protocol was used to provide adequate anesthetic depth and analgesia while reducing reliance on inhalant anesthesia. During the first surgical procedure, increases in HR and RR during surgical stimulation indicated insufficient nociceptive control and required rescue ketamine boluses. This finding emphasized the nociceptive intensity of the procedure and the need for close intraoperative assessment and supplemental analgesia when required. Prolonged surgical procedures and intraoperative nociceptive responses are also relevant peri-anesthetic considerations in pet rabbits (1). Close cardiorespiratory and temperature monitoring was therefore maintained throughout both surgical interventions. Because peri-anesthetic hypothermia is a recognized concern in rabbits, active warming and continuous temperature assessment were also considered important components of perioperative management (27). Intravenous lidocaine was selected as part of a multimodal analgesic strategy because continuous infusion at 100 μg/kg/min has previously been evaluated in rabbits and has been associated with reduced inhalant anesthetic requirements, postoperative analgesic benefit, and favorable effects on gastrointestinal motility after surgery (28, 29). Overall, the potential reconstructive benefit of fascia lata harvesting had to be balanced against the additional surgical and anesthetic time required in this geriatric and systemically compromised patient.

At follow-up, no recurrent herniation was detected clinically or by imaging. At 10 days, ultrasonography demonstrated continuity of the reconstructed abdominal wall without evidence of graft displacement or fluid accumulation. CT at three months and ultrasonography at six months confirmed maintenance of the repair and absence of recurrence. Because no biopsy or histopathological examination of the graft was performed, biological or histological integration cannot be confirmed, and terms such as “complete graft integration” should therefore be avoided.

To the authors’ knowledge, this is the first reported clinical use of a fascia lata autograft for recurrent inguinal hernia repair in a geriatric Netherland Dwarf pet rabbit. The favorable outcome in this case supports the feasibility of the technique when primary closure has failed and local tissues are compromised. However, conclusions regarding safety, long-term durability, or superiority over synthetic materials cannot be drawn from a single case. Although a variety of synthetic, composite, biologically active, and autologous reconstructive approaches have produced encouraging results experimentally (14–16, 18, 19), comparative clinical evidence in companion rabbits remains limited. Additional clinical cases with longer follow-up, objective assessment of donor-site function, and, where appropriate, histological or biomechanical evaluation would be required to better define the role of fascia lata autografts in abdominal wall reconstruction in rabbits. Such studies would contribute to the development of evidence-based surgical strategies for hernia repair in exotic companion mammals.

## 5 Conclusion

In this single clinical case, a fascia lata autograft provided tension-free reinforcement of a recurrent inguinal hernia after failure of primary closure in a geriatric rabbit with compromised tissue quality. No recurrence or clinically apparent graft-related or donor-site complications were observed during the six-month follow-up period. These findings suggest that autologous fascia lata may be a feasible reconstructive option in selected rabbits when primary closure is considered insufficient. However, conclusions regarding safety, long-term durability, recurrence rates, and broader clinical applicability cannot be drawn from a single case. Further clinical studies involving larger case series and longer follow-up are required to better define the role of this technique in exotic companion animal surgery.

## Data Availability

The original contributions presented in the study are included in the article/supplementary material, further inquiries can be directed to the corresponding authors.
